# Emergence of Recombinant Myxoma Virus in Algerian Farmed Rabbits: Molecular and Phylogenetic Evidence

**DOI:** 10.1155/tbed/9374702

**Published:** 2026-04-02

**Authors:** Samia Maziz-Bettahar, Laetitia Montacq, Cécile Caubet, Nicolas Gaide, Fatiha Yahouni-Slimani, Lynda Sahraoui, Stéphane Bertagnoli

**Affiliations:** ^1^ Clinical Department, Institute of Veterinary Sciences, University of Blida 1, Blida, Algeria, univ-blida.dz; ^2^ Laboratory of Animal Health and Production, Higher National Veterinary School of Algiers, Algiers, Algeria; ^3^ IHAP, ENVT, INRAE, University of Toulouse, Toulouse, France, univ-toulouse.fr; ^4^ Department of Producer Support and Relations with Livestock Sectors, Technical Institute of Livestock (ITELV), Baba Ali, Algiers, Algeria

**Keywords:** Algeria, detection, myxomatosis, rabbit farming, recombinant myxoma virus

## Abstract

Myxomatosis is a severe viral disease of lagomorphs that has recently seen the emergence of a recombinant myxoma virus (MYXV). Data from Africa remain scarce. Here we report the first confirmed detection and genomic characterization of a recombinant MYXV in Algeria. Two domestic rabbits from a backyard smallholding in El Affroun (Blida province) were presented on 19 October 2022 with classical signs of myxomatosis, including marked facial edema, severe mucopurulent blepharoconjunctivitis, and respiratory involvement. Necropsy and histopathology showed lesions typical of myxomatosis (epithelial hyperplasia with ballooning degeneration, intracytoplasmic inclusions, ulceration, stromal expansion by myxoma cells, and vascular hyperemia and leukostasis). MYXV DNA was detected in multiple tissues from both animals, and virus isolation on RK‐13 cells produced cytopathic effects (CPEs) by days 5–7 postinoculation. The Algerian MYXV sequence showed >99.9% nucleotide identity to the MYXV Toledo strain (GenBank Accession Number MK836424.1). Phylogenetic analysis placed the Algerian strain within the recombinant ha‐MYXV cluster, between earlier Spanish sequences (2018) and more recent Northern European sequences (2024), with the most recent common ancestor estimated around January 2017 (95% HPD July 2015–May 2018). These findings extend the known range of recombinant MYXV beyond Europe and highlight introduction risks at the pet–farm–wildlife interface in North Africa. We recommend strengthened vaccination of pet and farmed rabbits, pragmatic biosecurity in smallholdings, and targeted surveillance of hare populations to clarify sources and transmission routes.

## 1. Introduction

Myxomatosis is a major viral disease affecting both domestic and wild rabbits (*Oryctolagus cuniculus*), with significant consequences for animal health, biodiversity, and rabbit production. It is caused by myxoma virus (MYXV), a member of the genus *Leporipoxvirus* within the subfamily *Chordopoxvirinae* of the *Poxviridae* family [1]. In its classical form, the disease is characterized by proliferative cutaneous lesions with abundant myxoid matrix, particularly around the head and face, resulting in a typical swollen appearance. These signs are frequently accompanied by respiratory distress and blepharoconjunctivitis, and in severe cases, the disease often leads to death. This form is primarily transmitted by arthropod vectors, such as mosquitoes and fleas, which inoculate the virus through the skin [2]. In addition to the classical form, myxomatosis may also occur in a respiratory or amyxomatous form, in which cutaneous lesions are minimal or absent. The origin of this atypical form remains unclear, with hypotheses including viral mutations or the expression of a previously unrecognized latent form [3, 4].

Historically, myxomatosis was first described in 1896 in laboratory rabbits in Uruguay, where it manifested as a severe and fatal disease. The causative agent, later identified as MYXV, was found to circulate in natural host *Sylvilagus brasiliensis*, which serves as reservoir. In 1950, the virus was deliberately introduced into Australia as a biological control agent against invasive rabbit populations, followed by its introduction into France in 1952. These events triggered large‐scale epizootics, with mortality rates exceeding 90% in naïve rabbit populations. Over the decades, myxomatosis became enzootic in various regions, evolving into seasonal outbreaks with variable clinical severity [2, 5].

MYXV is a large enveloped virus with a brick‐shaped morphology (286 × 230 × 75 nm). The reference strain, Lausanne, has a genome composed of a linear double‐stranded DNA molecule of ~161.8 kilobases, featuring covalently closed hairpin termini and terminal inverted repeats (TIRs). It encodes 171 open reading frames (ORFs), including 12 duplicated within the TIRs. The central region of the genome contains highly conserved genes involved in replication and virion structure, while the terminal regions harbor genes associated with host range, immune modulation, and virulence [1, 6].

Until recently, infections with MYXV in hares were considered exceptional and limited to sporadic cases [7]. However, since 2018, outbreaks of myxomatosis have been reported in Iberian hares (*Lepus granatensis*) in Spain and Portugal [8, 9]. These outbreaks have been linked to a recombinant strain of the virus, designated ha‐MYXV (also referred to as MYXV‐To). Subsequently, confirmed cases were reported in European brown hares (*Lepus europaeus*) [10]. Importantly, although initially described in hares, the ha‐MYXV strain has also been detected in wild and domestic European rabbits [11, 12], indicating its capacity to infect both hares and rabbits.

Comparative genomic analyses indicate that ha‐MYXV diverges substantially from the reference strain of classic MYXV. It harbors an ~2.8‐kb insertion within M009L that encodes four novel ORFs homologous to M060, M061, M064, and M065, including an M064R‐like gene that may modulate host immune responses. In addition to this insertion, MYXV‐To shows major disruptions in three critical genes, including M009L, M036L, and M152R. These modifications may increase viral adaptability, facilitating cross‐species transmission and sustained infection in hare populations [13, 14].

Although myxomatosis is well documented in Europe and Australia, data from the African continent remain scarce. Only a few outbreaks have been reported. The most notable occurred in Egypt between autumn 2016 and spring 2017 across five governorates [15] and in Tunisia, where a reemergence was notified to World Organisation for Animal Health in 2006 after more than a decade without reported cases, with two outbreaks confirmed in domestic rabbits [16]. In Algeria, no official or published data have confirmed the occurrence or genetic characterization of MYXV. To our knowledge, this study provides the first molecular confirmation of myxomatosis in the country and shows that the isolated strain is genetically related to recombinant MYXV variants previously identified in Europe.

## 2. Materials and Methods

### 2.1. Animals and Case History

**Figure 1:**
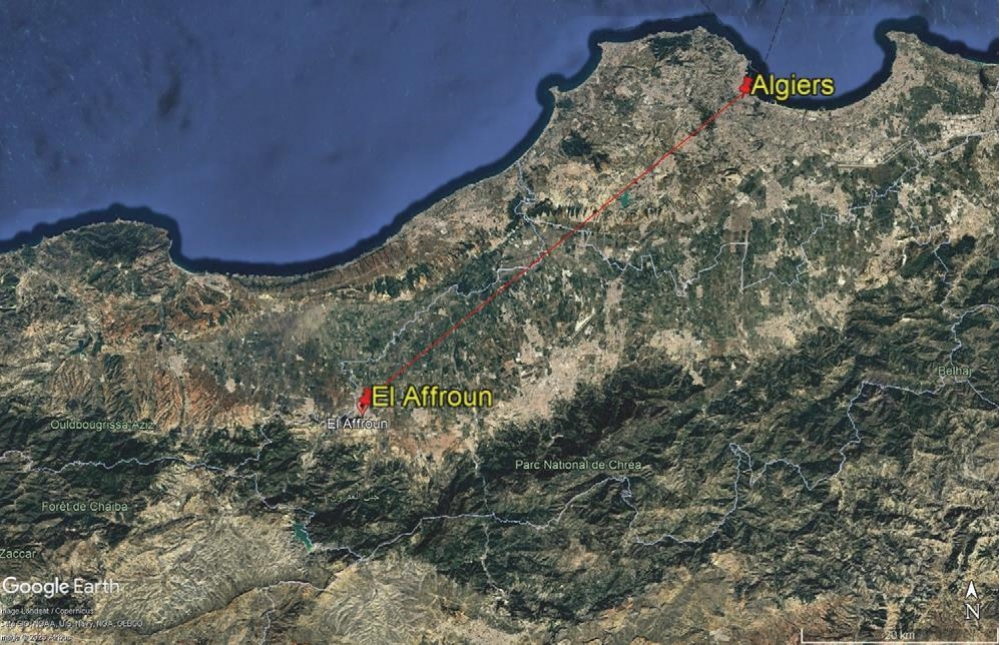
Geographical location of El Affroun (Blida Province), the origin of the sampled animals, located ~50 km southwest of Algiers, the capital of Algeria. Map generated using Google Earth Pro (Google LLC, Mountain View, CA, USA). 2025 Google.

Two domestic rabbits (one male and one female) were submitted on October 19, 2022, to the Research Laboratory for Animal Health and Production at the Higher National Veterinary School of Algiers for laboratory investigations. The animals originated from a small rural farm located in the El Affroun district (36°28′12.57"N, 2°37′32.67"E), Blida province, ~50 km southwest of Algiers, the capital city of Algeria (Figure 1). They belonged to a local farmed rabbit population raised under traditional backyard conditions. According to the breeder, the entire litter of six kits had been lost 1 week prior to submission.

### 2.2. Necropsy and Histopathological Analysis

A complete necropsy was performed on the female rabbit (Rabbit 2), admitted alive but euthanized at admission due to its severe condition. The male (Rabbit 1) had been dead for more than 24 h when received, showing marked autolysis. Lesions and significant findings were recorded. Tissue samples including lung, liver, kidney, spleen, testis, and eyelid were collected for laboratory analysis. Samples intended for virological and molecular analyses were stored at −20°C until further processing. For histological examination, tissue samples were fixed in 10% neutral‐buffered formalin, paraffin‐embedded, sectioned at 4 µm, and stained with hematoxylin and eosin (H&E).

### 2.3. Virology Isolation and Semi‐Purification

Virus isolation was performed using confluent monolayers of rabbit kidney (RK‐13) cells (ATCC, CCL‐37) maintained in Dulbecco’s Modified Eagle Medium (DMEM) supplemented with 10% fetal calf serum (FCS), penicillin (100 IU/mL), streptomycin (100 µg/mL), and antifungal agents. Two tissue samples were selected for inoculation; testicular tissue from Rabbit 1 and an eyelid fragment from Rabbit 2. Tissues were homogenized in DMEM using both manual and automated homogenization methods, then clarified by centrifugation. A volume of 500 µL of each supernatant was inoculated into RK‐13 monolayers cultured in 6‐well plates. Following a 1‐h adsorption at 37°C, the inoculum was removed, the cells were rinsed with phosphate‐buffered saline (PBS), and fresh supplemented medium was added. The cultures were incubated at 37°C in a humidified atmosphere containing 5% CO_2_ and observed daily for the appearance of cytopathic effects (CPEs) by phase contrast microscopy.

After 7 days of incubation, wells exhibiting the most pronounced CPEs by individual were selected. The contents of these wells were harvested and used to infect fresh RK‐13 cell monolayers seeded in 175 cm^2^ flasks. At 2 days postinoculation, cells were collected by centrifugation, and the resulting pellets were resuspended in 1 mL of PBS supplemented with a protease inhibitor cocktail (Complete Protease Inhibitor Cocktail, Roche). Cell suspensions were homogenized using a Dounce tissue grinder.

The homogenates were semi‐purified by ultracentrifugation through two successive sucrose cushions (36% w/v in PBS). For each purification step, 3.2 mL ultracentrifuge tubes were layered with 2 mL of 36% sucrose solution followed by 1 mL of homogenate (first run) or resuspended pellet (second run). Ultracentrifugation was performed using an Optima MAX‐E ultracentrifuge (Beckman Coulter) equipped with a TLA‐110 rotor at 36,000 rpm (52,980 × g; *k*‐factor: 139) for 60 and 90 min for the first and second runs, respectively. The final pellet was resuspended in 200 µL of PBS for DNA extraction for sequencing.

### 2.4. Molecular Analysis

#### 2.4.1. DNA Extraction and Real‐Time PCR (qPCR)

Tissue samples from the spleen, lung, and testicle of Rabbit 1 and the spleen and eyelid of Rabbit 2 were processed for viral DNA extraction using a commercial kit (Bio Basic Canada Inc.), following the manufacturer’s instructions. Detection of MYXV DNA was carried out using a TaqMan real‐time PCR assay targeting the M071L gene, with primers F‐M071LqPCR (5^′^‐GACATTTTAGCCTATGGGAAGAGTATGTA‐3^′^) and R‐M071LqPCR (5^′^‐AAGCAACGTCGTATCGTCCT‐3^′^), along with a fluorogenic probe (5^′^‐6‐FAM‐ACGCGCGTCAAAGATCCGAACA‐BHQ1‐3^′^) synthesized by Eurogentec (Liège, Belgium).

Each qPCR reaction was performed in a final volume of 20 µL, consisting of 10 µL of Master Mix (QIAGEN GmbH, Hilden, Germany), 0.6 µL of each primer, 0.2 µL of TaqMan probe, 6.6 µL of DNase‐free water, and 2 µL of DNA template. All reactions were run in duplicate to ensure reproducibility. Each run included a negative control (DNase‐free water) and a positive control (virus DNA diluted 1:100). Amplification was carried out using a real‐time PCR thermocycler under the following cycling conditions: initial denaturation at 95°C for 5 min, followed by 40 cycles of 95°C for 5 s and 58°C for 20 s.

#### 2.4.2. Next‐Generation Sequencing (NGS)

Semi‐purified viral material from both rabbits was resuspended in 200 µL PBS and subjected to nucleic acid extraction using the NucleoMag Viral RNA/DNA Isolation Kit (Macherey‐Nagel, Germany). To identify the sample best suited for NGS, MYXV load was quantified by qPCR and total DNA concentration was measured with the Qubit High‐Sensitivity dsDNA Assay Kit (Invitrogen, USA). At a 1:100 dilution, Ct values were 19.4 for Rabbit 1 and 18.7 for Rabbit 2, with total DNA concentrations of 12 and 29 ng/µL, respectively. Because the Rabbit 1 extract contained a slightly higher proportion of viral genomic material relative to total DNA, it was selected for Illumina sequencing (INVIEW Virus service, Eurofins Genomics, Germany).

To differentiate the terminal regions, the TIRs were amplified separately by PCR, and the resulting amplicons were sequenced using Oxford Nanopore Technology via the Linear Amplicon ONT service (Eurofins Genomics, Germany). PCRs were conducted using PrimeStar Max 2X Master Mix (Takara) with 200 nM of each primer. For amplification of the left TIR, primers M0005‐F and M009‐R, as described by Rossini et al. [17], were used. For the right TIR, primer M0005‐F was combined with a custom‐designed primer, M153‐Fbis.

PCR cycling conditions were as follows: initial denaturation at 98°C for 3 min; 35 cycles of denaturation at 98°C for 10 s, annealing at 55°C for 30 s, and extension at 72°C for 10 min; followed by a final extension at 72°C for 10 min.

**Table:** 

Primer name	Primer sequence	Position(s) on MK836424.1 sequence
M0005‐F	ACGCGGAAGTCTGCCTATTT	236 → 255/164,344 → 164,325
M009‐R	ACGAGAGATACGCTGAAGAAC	15,314 → 15,294
M153‐Fbis	TTCTCGTCTCTGCGTCCATG	151,755 → 151,774

#### 2.4.3. Whole Genome Assembly (Bioinformatic Analysis)

Unless otherwise specified, default parameters were applied for all bioinformatic analyses. Illumina reads from the raw DNA extract were first mapped to the host genome (*O. cuniculus*, GenBank Accession Number OZ175184.1) using BWA‐MEM [18]. Reads that did not map to the host genome were subsequently assembled de novo using the SPAdes assembler [19]. De novo assemblies of TIR amplicons were generated by Eurofins Genomics. The main genomic contig and TIR sequences were manually merged based on overlapping regions to reconstruct a near‐complete genome. The most similar published sequence was identified via BLAST and used to recover the terminal sequences (flanking the TIR amplicons) by generating a consensus with iVar [20]. To assess assembly robustness and investigate the presence of potential viral mixtures, sequencing reads were remapped to the complete genome sequence, visually inspected, and subjected to variant calling using iVar.

#### 2.4.4. Phylogenetic Tree Construction

To construct a time‐calibrated phylogenetic tree, all complete MYXV genomes available in GenBank were retrieved, excluding laboratory strains and sequences with unknown collection dates. Sequences were aligned using MAFFT [21].

Molecular dating was performed with BEAST2 [22] using a Bayesian Markov chain Monte Carlo (MCMC) framework. To account for differing evolutionary rates across the genome, the alignment was divided into three partitions: a central region and two flanking regions containing large insertion/deletion events. Analyses used a lognormal relaxed molecular clock [23], a coalescent Bayesian skyline tree prior [24], and three monophyletic constraints (Chilean samples; Finnish samples; ha‐MYXV sequences). Each partition was modeled with HKY substitution parameters [25], unequal base frequencies, a proportion of invariant sites, and gamma‐distributed rate heterogeneity, while sharing a single clock and tree model.

MCMC chains were run for 200 billion steps, discarding the first 10% as burn‐in. Three independent runs were performed to confirm convergence and reproducibility, which were assessed with Tracer v1.7.2 [26].

## 3. Results

### 3.1. Clinical, Necropsy and Histopathological Findings

The Rabbit 1 showed marked facial edema associated with severe bilateral congestive and edematous blepharoconjunctivitis and moderate mucopurulent discharge, leading to complete closure of the eyes (Figure 2A). The fur appeared unkempt and soiled, suggesting a generally poor condition. The Rabbit 2 displayed more pronounced respiratory involvement, including serous nasal discharge, dyspnea, and prostration. Ocular signs were also prominent, with intense conjunctival hyperemia, swollen eyelids, and mucopurulent blepharoconjunctivitis partially obscuring the eyes (Figure 2B).

Figure 2Clinical appearance of the two rabbits affected by myxomatosis. (A) Male rabbit (Rabbit 1), eyelids are closed and thickened due to severe edema, hyperemia, and purulent discharge. The fur appears dirty, suggestive of poor general condition. (B) Female rabbit (Rabbit 2) exhibiting purulent conjunctivitis, pronounced eyelid edema, and clear nasal discharge sticking to the surrounding nasal hair, accompanied by a hunched posture, signs of prostration, and poor body condition.

**Figure:**
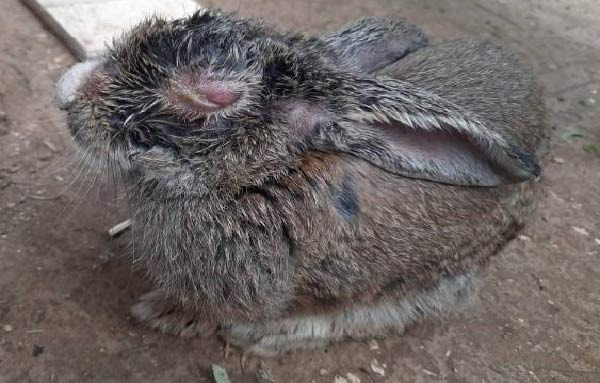
(A)

**Figure:**
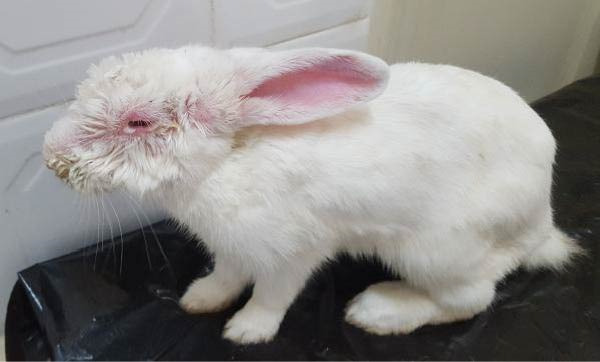
(B)

At necropsy of Rabbit 2, the main findings included poor body condition and severe congestive‐edematous and mucopurulent blepharoconjunctivitis that had been observed while the animal was alive. Additionally, sinuses were filled with white foamy translucid material and consistent with bilateral serous sinusitis. Nonspecific acute multifocal pulmonary congestion and hemorrhages were observed. The spleen, liver, and kidneys showed no significant macroscopic abnormalities. The digestive tract, including the colon, cecum, and small intestine, appeared within normal limits, except that the intestinal Peyer’s patches exhibited focal pinpoint red to brown discolorations.

Histological examination revealed diffuse, severe, palpebral expansion and thickening due to both proliferative and inflammatory processes involving both palpebral epithelium and stroma (Figure 3A). Surface and follicular epithelium showed epithelial hyperplasia, ballooning degeneration, and occasional intracytoplasmic eosinophilic viral inclusion bodies (Figure 3B). Multifocally, epidermis was disrupted by epithelial necrosis, superficial erosion, and ulceration (Figure 3C). The underlying stroma was expanded by numerous large spindle to stellate cells (myxoma cells), exhibiting large vesicular nuclei, indistinct nucleoli, and rare mitotic figures. The interstitium was additionally infiltrated by moderate numbers of heterophils, and blood vessels showed signs of hyperemia with endothelial reactional hypertrophy and heterophilic leukostasis (Figure 3D).

Figure 3Histological findings in Rabbit 2. Tissue sections are stained with hematoxylin and eosin stain (H&E). (A) Diffuse palpebral expansion of both epithelial and mesenchymal compartments, scale bar = 500 µm. (B) Ballooning degeneration (asterisk) and intracytoplasmic eosinophilic inclusion bodies (arrowhead) in follicular epithelial cells, scale bar = 50 µm. (C) Epidermal and follicular degeneration and necrosis (arrowhead), scale bar = 100 µm. (D) Vascular hyperemia, leukostasis (asterisk), myxoma cells (arrowhead), scale bar = 50 µm.

**Figure:**
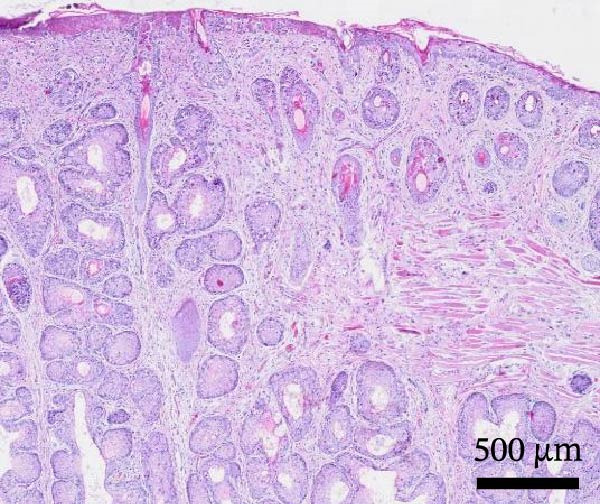
(A)

**Figure:**
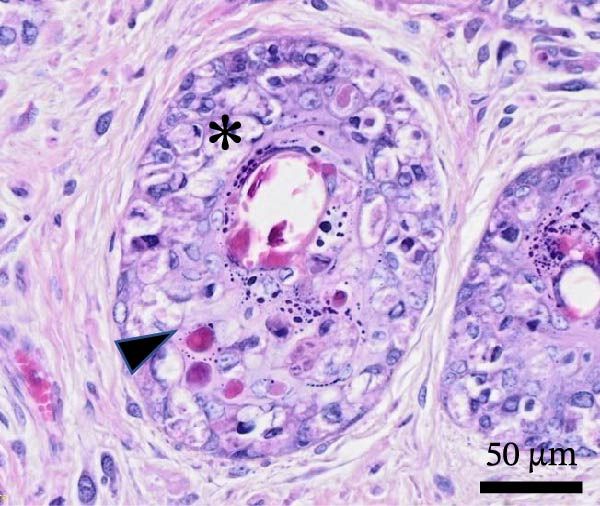
(B)

**Figure:**
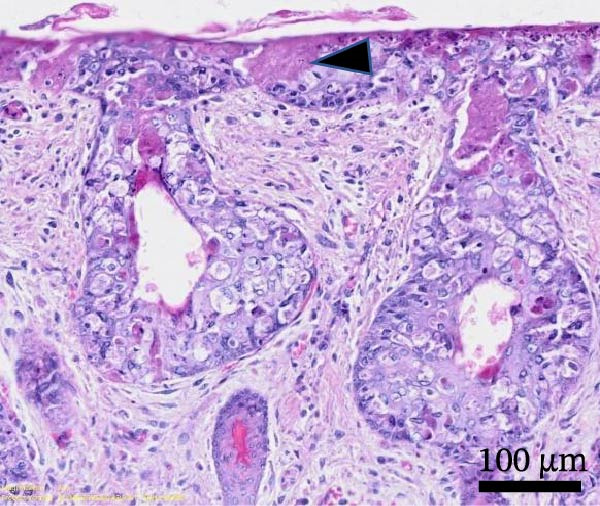
(C)

**Figure:**
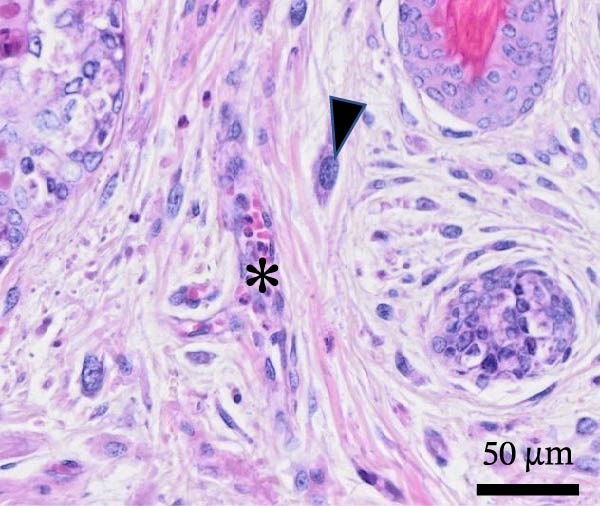
(D)

### 3.2. MYXV Detection

All tissue samples tested by qPCR were positive for MYXV. The virus was detected in the spleen, lung, and testicle of Rabbit 1, as well as in the spleen and eyelid of Rabbit 2. The lowest cycle threshold (Ct) values were observed in the eyelid of Rabbit 2 (Ct = 12.9) and in the testicle of Rabbit 1 (Ct = 15.6). In contrast, higher Ct values, reflecting lower viral loads, were obtained from the lung and spleen samples of both rabbits (Ct = 20.5 and Ct = 25.3, respectively). No amplification was detected in the negative controls, confirming both the specificity of the assay and the absence of cross‐contamination.

### 3.3. Virology Isolation

CPEs were observed at the first passage by day 5 postinfection in RK‐13 cell cultures inoculated with the eyelid sample from Rabbit 2 (Figure 4A). In cultures inoculated with the testicular sample from Rabbit 1, CPE were observed at the second passage. Infected cells showed characteristic changes, including rounding, detachment, and lysis, indicating active viral replication.

Figure 4Isolation of myxoma virus on RK‐13 cells. (A) Cytopathic effect in RK‐13 cells infected with a myxoma virus isolate from the eyelid sample of Rabbit 2 at day 5 postinfection. (B) Monolayer of RK‐13 cells, noninfected control (100x).

**Figure:**
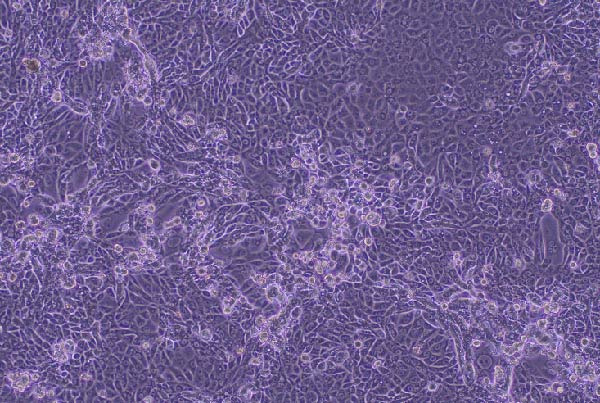
(A)

**Figure:**
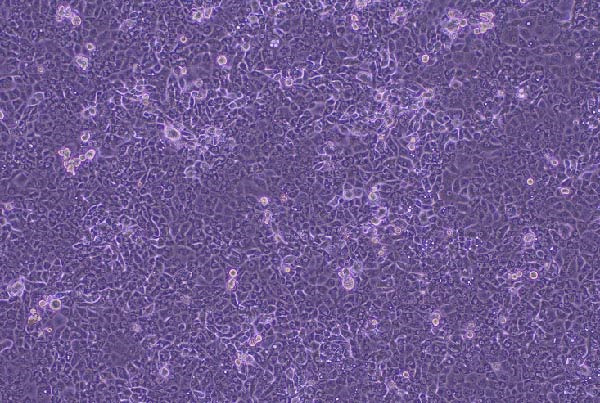
(B)

### 3.4. Genetic Analysis

Illumina sequencing generated a total of 7,494,444 reads, corresponding to 2,248,333,200 bases. Approximately 88% of these reads mapped to the *O. cuniculus* genome and were excluded from downstream analysis. De novo assembly of the unmapped reads yielded a single predominant contig of 141.5 kb, with an average coverage depth of ~800×.

Sequencing of the TIR’s amplicons produced 1116 reads for the left TIR and 2173 reads for the right TIR, with mean coverage depths of ~120× and 380×, respectively. TIR contigs and the main genomic contig were manually merged based on overlapping regions to reconstruct the near‐complete genome. No discrepancies were observed in the overlapping sequences, except for the presence of an additional A/T within a homopolymeric stretch in the TIR contigs. This variation was attributed to a known sequencing artifact associated with Oxford Nanopore Technology and was excluded from the final assembly.

The complete viral genome was highly similar to the MYXV Toledo strain (GenBank Accession Number MK836424.1), with 164,075 out of 164,092 positions identical, corresponding to >99.9% nucleotide identity. The terminal regions, flanking TIRs amplicons, were retrieved by mapping Illumina reads against MYXV Toledo strain left extremity (identical to the right one) and did not show any difference.

Among the observed differences, a single T insertion in the ha‐m065L gene (encoding the recombinant poxvirus poly(A) polymerase subunit) introduced a frameshift mutation, resulting in a predicted truncated protein. In addition, two codon insertions were detected in the ha‐m064L gene, leading to the elongation of lysine and aspartic acid repeats. Other point mutations neither caused frameshifts nor altered protein lengths.

Variant calling revealed a highly homogeneous viral population, with only four positions showing variant frequencies exceeding 5%, ranging from 7% to 12%.

**Figure 5:**
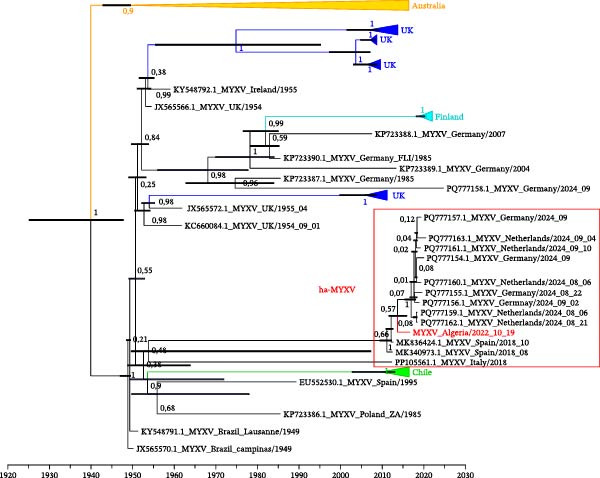
Time‐calibrated Bayesian phylogeny of myxoma virus (MYXV) inferred from 137 complete genome sequences partitioned into three subsets and analyzed under HKY substitution models in BEAST2 with a relaxed lognormal molecular clock. Posterior probabilities are shown at key nodes, and the bars on the nodes represent the 95% highest posterior density (HPD) intervals for the estimated dates. The Algerian recombinant ha‐MYXV sequence (2022) is highlighted in red. Sequences were obtained from *Oryctolagus cuniculus* unless otherwise indicated:  ^∗^ (*Lepus europaeus*), # (*Lepus granatensis*), and ° (*Lepus corsicanus*). The phylogeny illustrates the temporal and geographic structuring of MYXV lineages, with the Algerian sequence positioned within the ha‐MYXV cluster.

This Algerian MYXV sequence represents the first recombinant ha‐MYXV detected outside Western Europe. The placement of ha‐MYXV sequences in the time‐calibrated phylogenetic tree (Figure 5) suggests a common and recent origin, with the oldest sequences (Spain, 2018) at the base of the cluster, the Algerian sequence (2022) in the middle, and the most recent sequences (Germany and the Netherlands, 2024) at the tips. The most recent common ancestor of ha‐MYXV viruses is estimated to date back to January 2017 (95% HPD: July 2015–May 2018), indicating recent dissemination events. Precisely dating the recombination event is currently impossible, as posterior support for deeper branches is weak and the time estimates are broad. We can only conclude that the event occurred after the introduction of MYXV into Europe and before 2017.

## 4. Discussion

Myxomatosis is a major viral disease of domestic and wild lagomorphs, characterized by high morbidity and mortality, and it poses a serious threat to both rabbit production and wildlife. In recent decades, a recombinant MYXV has been reported, particularly in Europe, where it has been detected in Iberian hare (*L. granatensis*), European brown hare (*L. europaeus*), and domestic rabbit populations, raising significant epidemiological concern [9–12, 27]. In Africa, information on MYXV circulation is scarce; to our knowledge, recombinant MYXV has not been reported previously. Our findings constitute the first molecular evidence in Algeria, extending its known distribution beyond Europe.

In our study, the episode involved a small family‐owned rabbitry (one breeding pair and a litter of six kits). The lack of vaccination in adults rendered them fully susceptible to infection; the total mortality of the litter underscores the severity of the disease in young rabbits lacking maternal immunity at birth [28]. The farm was located near a temporary watercourse (wadi), an environment conducive to mosquito proliferation, which are recognized as mechanical vectors of MYXV [2]. The generally low biosecurity in small‐scale Algerian rabbitries [29] likely facilitated the introduction and subsequent spread of the virus.

The clinical and pathological findings observed in the two affected rabbits were consistent with myxomatosis, including severe ocular involvement, marked facial edema, respiratory signs, and a general deterioration of body condition. Histopathological examination revealed epithelial hyperplasia, ballooning degeneration, foci of necrosis, intracytoplasmic inclusion bodies, and characteristic myxoma cells. These findings are in agreement with previous descriptions reported in European rabbits as well as in hares, particularly *L. granatensis* (Iberian hare) and *L. europaeus* (European brown hare), and confirm that, despite the recombinant nature of the detected virus, the clinical and histological presentation remains indistinguishable from that of usual myxomatosis [8, 12, 27].

Sequencing of semi‐purified isolates yielded a complete MYXV genome showing >99.9% nucleotide identity with the MYXV Toledo strain, confirming that the Algerian isolate belongs to the recombinant ha‐MYXV phylogenetic group described in Europe. The data are consistent with an introduction from Europe. In this context, the recent development of pet rabbit husbandry, whose breeding stock is generally imported from Europe, together with the commercial exchanges that Algeria maintains with Western countries could represent a plausible route for the introduction of recombinant MYXV, as has been proposed for RHDV2 [30].

The population of domestic rabbits in Algeria is growing rapidly, driven in particular by the rise of pet rabbits in a context where vaccination coverage against myxomatosis remains limited. This combination, together with low biosecurity levels in smallholdings, increases the risk of viral introduction and spread. In parallel, as in other Mediterranean countries, Algeria harbors wild lagomorph populations that occupy vast areas and constitute a game resource: two hare species, *Lepus capensis*, widely distributed from the north to the southern margins, and *Lepus saxatilis*, rarer in the southeast [31]. This wildlife–livestock–pet interface creates opportunities for circulation and maintenance of MYXV. In our study, the recombinant myxomatosis virus, initially described in hares, was detected in two farmed rabbits, confirming its ability to infect this species. This observation highlights the potential circulation of the recombinant within domestic populations. However, the role of the hare, the historical host of the recombinant, in transmission dynamics remains to be explored. Investigating hare populations could provide essential insights into the origin, persistence, and spread of the virus across Algeria.

## 5. Conclusion

This study reports, to our knowledge, the first detection and molecular characterization of a recombinant MYXV in farmed rabbits in Algeria. This observation suggests the existence of multiple introduction pathways, plausibly linked to the pet‐rabbit trade and/or contacts at the wildlife–livestock interface. It underscores the need for (i) epidemiological surveillance of wild lagomorphs, particularly hare populations; (ii) improved vaccination coverage, notably for pet rabbits and in farm holdings; and (iii) a pragmatic strengthening of biosecurity measures in smallholder/backyard farms, taking into account real‐world implementation constraints.

## Author Contributions

**Samia Maziz-Bettahar and Laetitia Montacq:** writing – original draft. **Samia Maziz-Bettahar**, **Fatiha Yahouni-Slimani, and Lynda Sahraoui:** data collection. **Samia Maziz-Bettahar**, **Laetitia Montacq, Cécile Caubet, and Nicolas Gaide:** investigation (necropsy/sampling, cell culture, molecular testing, histopathology). **Laetitia Montacq:** formal analysis (bioinformatics). **Nicolas Gaide:** manuscript revision. **Stéphane Bertagnoli:** supervision and manuscript revision.

## Funding

This work received no external funding.

## Disclosure

Molecular analyses were performed at the National Veterinary School of Toulouse (ENVT), within the Host–Pathogen Interactions research unit.

## Ethics Statement

Ethical review and approval were not required for this study, as all samples used for diagnostic purposes were collected postmortem from deceased animals. The study was conducted in accordance with local legislation and institutional requirements.

## Conflicts of Interest

The authors declare no conflicts of interest.

## Data Availability

Data produced in this study are available at GenBank (www.ncbi.nlm.nih.gov/genbank/) under Accession Number PX436186.1
